# Prevalence, incidence, and years-lived with disability due to oral disorders in Brazil: an analysis of the Global Burden of Disease Study 2019

**DOI:** 10.1590/0037-8682-0284-2021

**Published:** 2022-01-28

**Authors:** Fernando Neves Hugo, Jordan A Bailey, Caroline Stein, Amanda Ramos da Cunha, Betine Pinto Moehlecke Iser, Deborah Carvalho Malta, Jessye Melgarejo do Amaral Giordani, Juliana Balbinot Hilgert, Lucas Guimarães Abreu, Nicholas J Kassebaum

**Affiliations:** 1 Universidade Federal do Rio Grande do Sul, Departamento de Odontologia Preventiva e Social, Porto Alegre, RS, Brasil.; 2 University of Washington, Institute of Health Metrics and Evaluation, Seattle, WA, USA.; 3 Universidade Federal do Rio Grande do Sul, Programa de Pós-Graduação em Epidemiologia, Porto Alegre, RS, Brasil.; 4 Universidade Federal do Rio Grande do Sul, Programa de Pós-Graduação em Odontologia, Porto Alegre, RS, Brasil.; 5 Universidade do Sul de Santa Catarina, Programa de Pós-Graduação em Ciências da Saúde, Tubarão, SC, Brasil.; 6 Universidade Federal de Minas Gerais, Departamento de Saúde Materno-Infantil e Saúde Pública, Belo Horizonte, MG, Brasil.; 7 Universidade Federal de Santa Maria, Departamento de Estomatologia, Santa Maria, RS, Brasil.; 8 Universidade Federal de Minas Gerais, Departamento de Saúde Bucal da Criança e do Adolescente, Belo Horizonte, MG, Brasil.

**Keywords:** Oral health, Global Health, Global Burden of Disease, Dental caries, Periodontitis, Tooth loss

## Abstract

**INTRODUCTION:**

Epidemiological surveys revealed that Brazil has a high burden of oral diseases. However, no prior study has reported estimates of untreated dental caries, periodontitis, and edentulism over a three-decade period. The objective of this study is to report the trends of prevalence, incidence, and years-lived with disability (YLDs) due to untreated dental caries in primary and permanent teeth, periodontitis, and edentulism in Brazil between 1990 and 2019.

**METHODS:**

Estimates of prevalence, incidence, and YLDs due to dental caries in primary and permanent teeth, periodontitis, and edentulism were produced for Brazil, by sex and age, between 1990 and 2019, using Dismod-MR 2.1, as part of the Global Burden of Disease Study 2019 (GBD 2019). Trends of oral disorders were analyzed using generalized linear regression models applying the Prais-Winsten method.

**RESULTS:**

Almost 100 million Brazilians presented at least one oral disorder in 2019, which was equivalent to a prevalence of 45.3%. All oral diseases combined ranked eighth among all causes of disability, causing more than 970,000 YLDs. Untreated dental caries in primary teeth were estimated to affect 13.5 million children, and untreated dental caries in permanent teeth affected more than 52 million people. Periodontitis affected 29.5 million people, and edentulism affected almost 22 million. The generalized linear regression models revealed a trend of stability of oral disorders between 1990 and 2019.

**CONCLUSIONS:**

The burden of oral diseases in Brazil is extremely high. Oral disorders, edentulism in particular, caused disability at levels that are comparable to other important chronic diseases.

## INTRODUCTION

Oral disorders, including dental caries, periodontitis, and edentulism are the most prevalent diseases of mankind, affecting more than 44.5% of the global population in 2019, according to the last report from the GBD-2019^1^. Brazil, in particular, has experienced a high burden of oral diseases. The substantial disability caused by oral diseases^2^ result in pain, sepsis, lost school days, decreased work productivity^3^ and overall worse quality of life and wellbeing^4^. Also, its economic impact should not be neglected, with direct treatment costs of dental diseases estimated to correspond to 4.6% of all health expenditure, globally^5^. While the last National Oral Health Survey, carried out in 2010, revealed a declining trend in dental caries experience in 12-year-old children, the mean number of untreated caries was still high among individuals in this and other age groups, with persisting inequalities in the distribution of the disease. The prevalence of edentulism has also remained extremely high, above 50%, in older adults^6^.

More than a decade has passed since the last Brazilian oral health survey. The newest national survey that had been planned to take place in 2020 has now been postponed until the end of the COVID-19 pandemic. In the scenario that follows, the use of estimates from the GBD-2019^1^ represents an excellent opportunity to analyze changes in oral disease burden in Brazil over a period of three decades. The analysis of changes in estimates may also help to inform policies such as Brazil’s National Oral Health Policy of 2004, which resulted in a meaningful expansion in the coverage of primary dental care^7^.

The GBD-2019^1^ is currently the most comprehensive epidemiological study of disease burden. Estimates provided by the GBD, which include prevalence, incidence, and years lived with disability (YLDs) due to Oral Disorders, including untreated caries in primary and permanent teeth, periodontitis, and edentulism, have the potential to provide key information to researchers, policymakers, and stakeholders in Brazil. However, since the beginning of publications based on the GBD study, no in-depth reports of oral disorders, dental caries in primary and permanent teeth, periodontitis, and edentulism estimates have been conducted for Brazil using these estimates.

The present study, therefore, sought to describe the trends of prevalence, incidence, and YLDs due to untreated dental caries in the primary and permanent teeth, periodontitis, and edentulism in Brazil, between 1990 and 2019^1^.

## METHODS

### Study design

The description of the methods to obtain estimates of prevalence, incidence, and YLDs due to dental caries in primary and permanent teeth, periodontitis, and edentulism is fully provided in the GBD capstone article. The estimates are reported for Brazil and the period between 1990 and 2019 and stem from the GBD-2019^1^. With each iteration of the GBD, the values of the entire time series (beginning in 1990) are re-estimated by cause of oral disorder, age, sex, and geography. All estimates are reported as percentages or rates and the respective 95% uncertainty intervals (i.e., UI - the range of values that are likely to include the correct estimate of prevalence, incidence or health loss for a disease). The GBD complies with the Guidelines for Accurate and Transparent Health Estimates Report (GATHER) statement^8^.

### Data

Data used to inform models for oral disorders were obtained from scientific articles and oral health surveys, and were updated at each new iteration of the GBD Study. For the GBD-2019, scientific articles obtained following a search of the Latin American and Caribbean Health Sciences Literature (LILACS) and the Scientific Electronic Library Online (SciELO), contributed with additional novel data to produce estimates, with a focus on the most recent period from 2014 to 2018. A total of 1,696 citations were identified in these searches, and after removal of duplicate hits, 147 were selected for full text review, and data from 77 new sources were extracted. Among the 77 new sources, 47 were from Brazil.

### Case definitions

Briefly, the case definition of edentulism is “any individual with zero remaining permanent teeth”. The case definition for untreated dental caries is “teeth with unmistakable coronal cavity at dentin level, root cavity in cementum that feels soft or leathery to probing, temporary restorations, or missing teeth extracted due to a caries lesion”. The reference definition for modelling caries was the presence of one or more teeth with current decay (for prevalence), whereas each additional carious tooth was counted as a separate incident event. The case definition for periodontitis is “loss of gingival tissue and alveolar bone destruction”. The reference definition for modelling data on periodontitis was the Community Periodontal Index of Treatment Needs (CPITN)=4, followed by Attachment loss >6 mm, and pocket depth >5 mm. The case definition for edentulism was “complete loss of natural teeth,” which could be assessed by self-reports or clinical examination.

### Modelling strategy

Estimates of prevalence, incidence, and YLDs due to dental caries in primary and permanent teeth, periodontitis, and edentulism were produced for Brazil by year, sex, and age. Dismod-MR 2.1 was used.

For all oral disorders, mortality was set at zero. For dental caries in primary teeth, models assumed zero incident cases in infants under one year of age and similar zero incident dental caries in primary teeth from age 11 onward. Disease estimates for untreated caries in primary teeth were produced for children between the ages of 0.5 and 12 years. For dental caries in permanent teeth, the models assumed zero incident cases in children under five years of age, with estimates for the population aged between 6 years and over. Incidence and prevalence of periodontitis were assigned to be zero until age eight, as periodontal disease is largely considered to be a disease of adulthood. Incidence of periodontitis was allowed to rise beginning at age nine, based on the youngest age at which there was a non-zero-point estimate for prevalence in the dataset. For edentulism, as one would expect for an irreversible condition, remission was set at zero for all ages. Incidence and prevalence were assigned to be zero during childhood. Incidence was allowed to rise beginning at age 15, which was chosen based on the age at which the permanent teeth are expected to have fully erupted in all individuals.

YLDs were calculated by multiplying frequency (prevalence), severity (disability weight), and duration of each oral disorder. The definition of disability associated with symptomatic dental caries is “this person has a toothache, which causes some difficulty to eat.” The definition of disability associated with symptomatic periodontitis is “bad breath, a bad taste in the mouth, and gums that bleed a little from time to time, but this does not interfere with daily activities.” The definition of disability used for symptomatic edentulism is “great difficulty in eating meats, fruits, and vegetables.” The disability weights are defined by a specific GBD survey^9^. The values calculated for dental caries, periodontitis, and edentulism were 0.01 (0.005-0.019), 0.007 (0.003-0.014), and 0.067 (0.045-0.095), respectively.

In all estimates, uncertainty was derived from 1,000 draws at every step of the computational process. Uncertainty comes from sampling error in data sources, the distribution of condition severity and disability weights, as well as model coefficients. The final estimates were calculated as the average across the 1,000 draws, with the 95% UI computed as percentiles 2.5 and 97.5 in the distribution^1^.

### Trend analysis

For the trend analysis, a generalized linear regression model applying the Prais-Winsten method was used. The dependent variable was the log-transformed measure, and the independent variable was the year. The estimate of the annual percent change (APC) - and its 95% confidence interval (CI_95%_) - was obtained by the calculations proposed by Antunes and Waldman^10^:

$$APC=\left(-1+10^{bl}\right)*100\%$$

$${CI}_{95\%}{}^{\text{lower }}=\left(-1+10^{\text{b}\text{1}\text{lower }}\right)*100\%$$

$${CI}_{95\%}{}^{\text{upper }}=\left(-1+10^{\text{b}\text{1}\text{upper }}\right)*100\%$$

Where *b1* is the regression coefficient, and *b1lower* and *b1upper* are the limits of its CI_95%_. The trend is an ascending trend if the APC and CI_95%_ are positive, whereas it is a declining trend if APC and CI_95%_ are negative and stationary if the CI_95%_ includes zero.

## RESULTS

It is estimated that almost 100 million Brazilians had at least one event regarding any oral disorder assessed in 2019, which was equivalent to 45.26% of the population. All oral diseases combined caused more than 970,000 YLDs. Untreated dental caries in primary teeth were estimated to affect 13.5 million children aged between 0.5 and 12 years, while untreated dental caries in permanent teeth affected more than 52 million Brazilians aged six years and over. Periodontitis affected more than 29.5 million Brazilians, and edentulism affected almost 22 million (Table 1).

The disability caused by these oral diseases was also not negligible. Edentulism alone caused more than 600,000 YLDs, followed by periodontitis, with almost 194,000 YLDs. Untreated caries in permanent teeth caused more than 51,000 YLDs and approximately 5,000 YLDs in primary teeth (Table 1).

**TABLE 1:** Estimates of prevalence, incidence, and Years-Lived with Disability (YLD) and the respective 95% uncertainty intervals (UI) due to oral disorders in Brazil, 2019.

	Prevalence		Incidence		YLDs	
	Number of cases	Per 100 (age-	Number of Cases	Per 100 (age-	Number of years	Rate (per 100,000)
	(in millions)	standardized)	(in millions)	standardized)	(in thousands)	(age-standardized)
Oral disorders	99,925	45.26	119,578	57.47	972,705	409.7
	(91,838-108,129)	(41.53-48.99)	(107,055-132,260)	(50.76-60.3)	(600,806-1,482,505)	(253.55-622.58)
Untreated dental caries in primary teeth	13,513	8.34	28,670	17.45	5,189	3.2
	(10.871-16.087)	(6.68-9.91)	(19,333-37.981)	(11.82-23.29)	(2,230-10,804)	(1.37-6.68)
Untreated dental caries in permanent teeth	52,130	22.99	86,665	38.29	51,312	22.66
	(45.031-60.071)	(19.81-26.46)	(77,687-95,635)	(34.01-42.43)	(23,299-98,928)	(10.25-43.58)
Periodontitis	29,639	11.97	2,764	1.14	193,800	78.25
	(21,450-38,106)	(8.68-15.42)	(2,203-3,325)	(0.91-1.38)	(74,383-424,741)	(30.01-170.47)
Edentulism	21,880	9.19	1.450	0.59	601,158	252.17
	(17,614-27,089)	(7.44-11.33)	(1,197-1,697)	(0.49-0.68)	(383,072-877,957)	(161.16-366.66)

**FIGURE 1:**
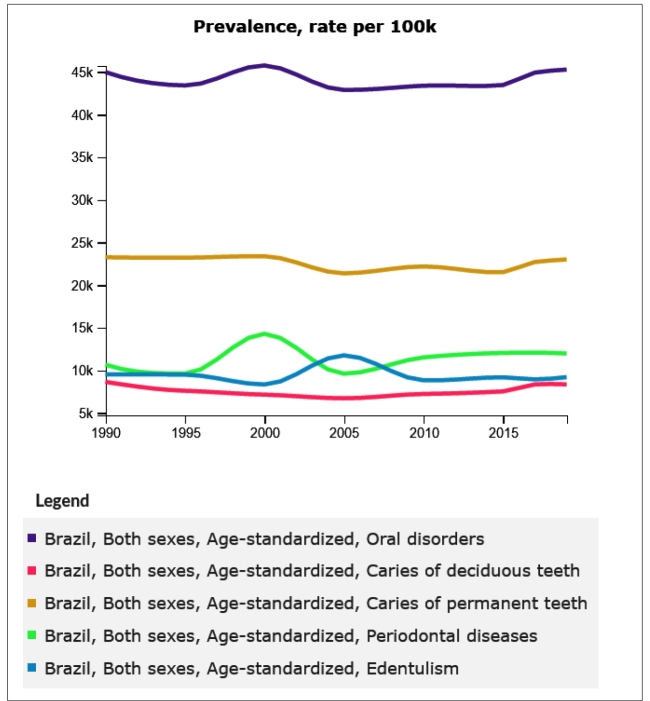
Trends of age-standardized prevalence of oral disorders, untreated dental caries in primary and permanent teeth, periodontitis, and edentulism in Brazil, 1990 to 2019.

**FIGURE 2:**
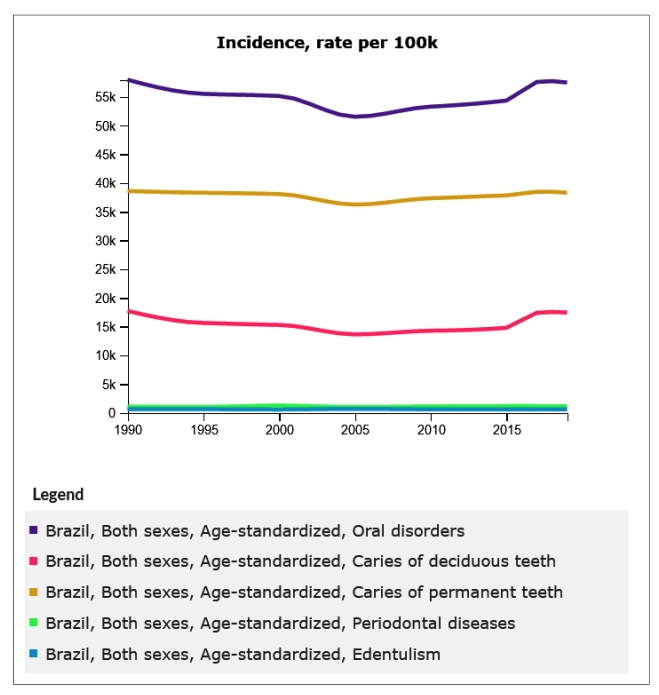
Trends of age-standardized incidence of oral disorders, untreated dental caries in primary and permanent teeth, periodontitis, and edentulism in Brazil, 1990 to 2019.

**FIGURE 3:**
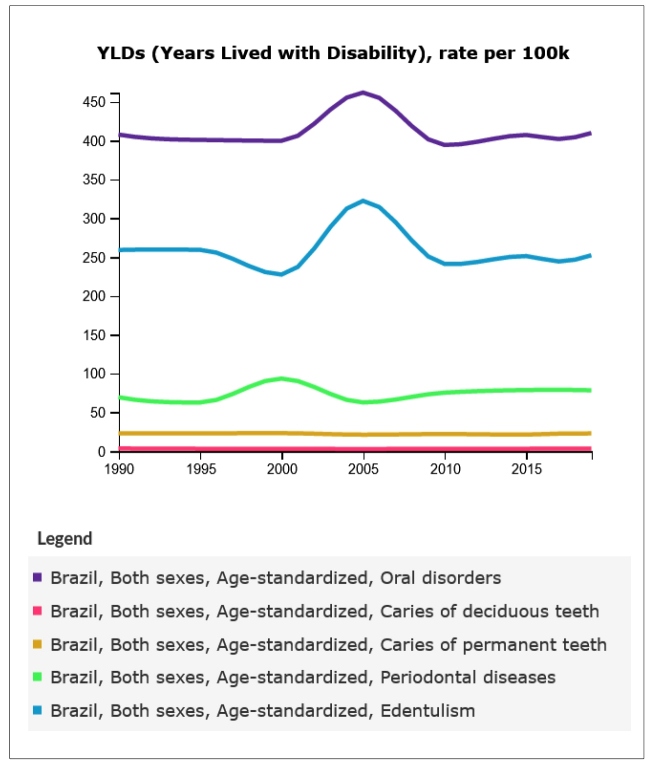
Trends of Years-Lived with Disability (YLDs), (all ages, rates per 100,000) due to oral disorders, untreated dental caries in primary and permanent teeth, periodontitis, and edentulism in Brazil, 1990 to 2019.

Though the APCs of prevalence, incidence, and YLDs of untreated dental caries in primary and permanent teeth and edentulism were negative, and the APCs of periodontitis disorders were positive, the trend analysis revealed stability over the three-decade period analyzed in this study. This is reflected in the fact that the APCs of prevalence, incidence, and YLDs of Oral Disorders all had values close to zero over the studied period. Finally, APCs in YLDs were of small magnitude, with the exception of periodontitis, with the generalized linear regressions also revealing trends of stability over time (Table 2). Graphs with trends of prevalence, incidence, and YLDs due to oral disorders, untreated dental caries in primary and permanent teeth, periodontitis, and edentulism are shown in Figures 1, 2, and 3. In general, these show a trend of stability over time, with the exception of the prevalence of periodontitis and edentulism and YLDs, respectively, in the period between 2003 and 2005.

**TABLE 2:** Percentage change in estimates of prevalence, incidence, and Years-Lived with Disability (YLD) and their respective 95% Confidence Intervals (UI) due to oral disorders in Brazil, between 1990 and 2019.

	Prevalence			Incidence			YLDs		
	APC(CI_95%_)^a^	Trend	% Change 1990-2019^b^	APC(CI_95%_)^a^	Trend	% Change 1990-2019^b^	APC(CI_95%_)^a^	Trend	% Change 1990-2019^b^
Oral disorders	0.01(-0.21;0.24)	↔	0.66	-0.03(-0.40;0.34)	↔	-0.83	0.02(-0.41;0.45)	↔	0.48
Untreated caries in primary teeth	-0.12(-0.85;0.61)	↔	-3.45	-0.05(-0.96;0.86)	↔	-1.45	-0.12(-0.84;0.61)	↔	-3.32
Under 5 years^c^	-0.09(-1.09;0.91)	↔	-2.70	-0.22(-1.22;0.80)	↔	-6.15	-0.09(-1.09;0.92)	↔	-2.56
5-9 years^c^	-0.16(-0.67;0.36)	↔	-4.52	0.02(-0.92;0.96)	↔	0.83	-0.15(-0.66;0.36)	↔	-4.41
5-14 years^c^	-0.25(-0.91;0.41)	↔	-7.10	-0.07(-1.09;0.96)	↔	-1.77	-0.25(-0.90;0.41)	↔	-7.00
Untreated caries in permanent teeth	-0.07(-0.38;0.25)	↔	-1.13	-0.03(-0.21;0.15)	↔	-0.73	-0.06(-0.36;0.25)	↔	-0.85
Periodontitis	0.43(-0.61;1.49)	↔	12.36	0.24(-0.34;0.83)	↔	6.69	0.45(-0.61;1.51)	↔	12.88
Edentulism	-0.11(-0.96;0.74)	↔	-3.41	-0.09(-0.54;0.36)	↔	-3.03	-0.09(-0.95;0.78)	↔	-2.64

^a^annual percent change (APC) of the rate per 100,000; ^b^percentage of change - comparing the years 1990 and 2019 - of the rates; ^c^APC and % change based on measures without age adjustment; ↔: stationary trend

Age-standardized prevalence and incidence for oral disorders in male and female individuals were similar, with the exception of the prevalence of periodontitis, for which male individuals showed a higher prevalence than did their female peers (13.05 vs. 11.01, respectively) and edentulism, for which female individuals showed a higher prevalence than did their male peers (10.64 vs. 7.51, respectively). Both differences, however, were not significant. This resulted in differences in terms of YLDs, with female individuals experiencing much more disability than male individuals (477.23 vs. 366.13, respectively). Additional results of prevalence, incidence, and YLDs by sex are presented in Supplementary Table 1.

## DISCUSSION

This study carried out a comprehensive description of estimates of prevalence, incidence, and YLDs due to oral disorders in Brazil over a three-decade period. The results reported herein are unprecedented, considering the extent and scope of the GBD-2019 and the fact that oral disorders are the most common disease worldwide, and Brazil is no exception^1^.

The burden of oral diseases in Brazil remains extremely high. Oral conditions affected almost 100 million people. Moreover, all oral diseases combined ranked eighth amongst all causes of disability in 2019^1^. The general trend of oral diseases was of stability, but population growth is likely to result in an overall increase in the number of people affected by oral diseases. Exceptions were the increases in prevalence (and consequently YLDs) due to periodontitis and edentulism around 2003 to 2005. These increases probably reflect the contribution of data from the National Oral Health Survey of 2003-04 that were incorporated into the models and the fact that it has results for many states in Brazil for which there was no previous data. The estimates of prevalence and incidence due to oral disorders in Brazil are similar to what has taken place globally, but Brazilians have experienced considerably more disease burden, as YLD rates are much higher than rates worldwide. This is because Brazil has displayed a prevalence of edentulism that is more than twice the Global prevalence^1^.

The Brazilian government carried out oral health surveys in 2003^11^ and 2010^6^ that provided nationally representative information on the prevalence of oral disorders, including caries in primary and permanent teeth, periodontitis, and edentulism. The results of these surveys, however, are not comparable with the estimates produced by the GBD-2019 because of differences in age groups assessed. The national surveys used the age groups recommended by the World Health Organization Pathfinder Survey methods^12^, while the GBD-2019 produced estimates for a wider range of ages, starting in early childhood and going up to old-old age.

Apart from differences in the prevalence and YLDs due to edentulism between male and female individuals, with the latter experiencing higher prevalence and more YLDs, no relevant differences by sex have been identified. Nevertheless, female individuals have higher overall YLDs. Such differences in terms of edentulism may be explained by the fact women use dental services more frequently^13^. This is particularly critical for older adults, for whom extraction of teeth and fabrication of dentures represented the only type of dental care available when they were younger^14^. This is somewhat different from the global estimates of oral disorders published by the GBD 2017 Oral Disorders Collaborators^15^, when no sex differences had been reported.

Similar to what has been reported in the capstone paper on oral disorders^15^, a large proportion of the Brazilian population has unmet needs of dental care^16^ ^,^ ^17^. While Brazil implemented a national oral health policy (Smiling Brazil) in 2004 that has since led to significant improvements in dental care funding and expenditure, access to primary dental care that is free of charge at the point of care^7^, population coverage by public dental care services, and provision of care by primary dental care is still limited^18^. Availability of consolidated dental care networks within Brazil’s public health system is also limited, meaning that a large proportion of the population that does not have resources to afford private dental care foregoes having their treatment needs met. This eventually leads to tooth extractions, the only type of dental care in places where no comprehensive dental care network has been constituted thus far^19^.

Limitations that have been acknowledged before also apply to the interpretation of the results of this study. These include the availability of primary data, even though this is not particularly relevant in the case of Brazil, where extensive sources of primary data are available. Another important aspect is related to the fact that some of the primary data used to generate the estimates have not been obtained using the preferred case definitions or measurement methods^1^. Future research that develops methods to harmonize data using different oral health indices is also needed to make estimates of oral disorders more meaningful and useful to researchers, stakeholders and policymakers.

In conclusion, the burden of oral diseases in Brazil is extremely high, and the analyses of trends over time revealed a stability in the prevalence, incidence and disability due to untreated caries of deciduous and permanent dentition, periodontitis and edentulism. Brazilians experience more YLDs in comparison to the world population, because of the high prevalence of edentulism (an age-standardized prevalence of edentulism in Brazil of 9.9% versus a global prevalence of 4.3%)^1^. YLDs due to oral disorders in the country are comparable, in terms of magnitude, with YLDs due to age-related hearing loss, blindness, and vision loss^1^. Although the country has significantly expanded access to dental care since the implementation of its National Oral Health Policy in 2004, the trends of oral diseases have remained stable, suggesting that many of the challenges that were existent are still in place. The fact that Brazil has more than 500 Dentistry undergraduate courses, the largest number of courses in any given country^20^, coupled with such a high burden of oral diseases, confirms the need to reform the oral healthcare delivery system towards prevention and oral health promotion in accordance with the population’s oral health needs^3^.
