# Supplementary material for: Prevalence, incidence, and years-lived with disability due to oral disorders in Brazil: an analysis of the Global Burden of Disease Study 2019
Source: Rev Soc Bras Med Trop. 2022 Jan 28;55(Suppl 1):e0284-2021. doi: 10.1590/0037-8682-0284-2021 (PMC9009423; doi:10.1590/0037-8682-0284-2021)
Supplement: Supplementary file 1 [file 1678-9849-rsbmt-55-s01-e0284-2021-supp1.pdf]

Supplementary Table 1. Estimates of prevalence, incidence, and Years-Lived with Disability (YLD) due to oral disorders in Brazil, by sex, 2019.

	Prevalence Per 100 (age-standardized)		Incidence Per 100 (age-standardized)		YLDs Rate (per 100,000) (age-standardized)	
	**Males**	**Females**	**Males**	**Females**	**Males**	**Females**
Oral disorders	44.68 (40.9-48.54)	45.77 (42.06-49.41)	57.13 (50.22-64.21)	57.77 (50.94-64.8)	366.13 (223.66-564.90)	447.23 (280.10-672.70)
Untreated dental caries in primary teeth	8.38 (6.73-9.97)	8.30 (6.6-9.82)	17.49 (11.86-23.03)	17.41 (11.83-22.99)	3.22 (1.39-6.71)	3.19 (1.38-6.67)
Untreated caries in permanent teeth	22,43 (19.23-25.89)	23.52 (20.32-27.03)	37.96 (33.6-42.23)	38.59 (34.38-42.7)	22.19 (9.96-42.23)	23.10 (10.51-43.98)
Periodontitis	13.05 (9.54-16-64)	11.01 (7.85-14.35)	1.17 (0.93-1.4)	1.11 (0.88-1.35)	85.56 (32.58-186.05)	71.67 (27.48-157.45)
Edentulism	7.51 (5.89-9.47)	10.64 (8.67-12.96)	0.52 (0.41-0.61)	0.66 (0.56-0.75)	205.72 (131.77-301.84)	292.07 (188.68-423.74)
